# Supplementary material for: Exploring the experiences of women of African, Caribbean and Mixed heritages to inform a music-based intervention for perinatal mental health in South East London: A qualitative study
Source: PLOS Ment Health. 2026 Mar 23;3(3):e0000477. doi: 10.1371/journal.pmen.0000477 (PMC13008082; doi:10.1371/journal.pmen.0000477)
Supplement: S1 Table — (DOCX) [file pmen.0000477.s001.docx]

| **Theme** | **Subtheme** | **Quotations** |
| --- | --- | --- |
| **Theme 1: Supportive mechanisms during the perinatal period** |  |  |
|  | **Emotional and practical support from family and friends** | *Focus Group 1:* "I was so grateful that my mum made me move into her house after I came back home from the hospital because I don't know how I would have coped. She was literally the one that forced food down my mouth." *Focus Group 1:* "I also had my second baby during the lockdown. And just the level of isolation after giving birth, where you're supposed to be surrounded by people who are helping you to navigate these things." *Focus Group 2:* "if you don't have a supportive partner, forget about family and friends, but if you don't have a partner in the house that can help you with certain situations, it's a tremendous burden and a struggle I find upon women." *Focus Group 4:* "There's no understanding there all the time, or you don't feel you get as much support because they're not in your position. Or you're judged for being in that position in the first place." *Focus Group 4:* "Knowing that there's people that you can call on when you're feeling low or you can just message them randomly and say, “hey are you alright? what are you doing”, and knowing that you'll get a reply within a certain amount of time, you're not just sitting there isolated on your own." |
|  | **Connection with wider community networks** | *Focus Group 1*: "hearing it from another mum who is going through either something similar or they’re having struggles of their own was quite refreshing, and also quite empowering in many ways. Like “we can get through this together” kind of thing, and that was absolutely lovely." *Focus Group 2:* "a few women, they don't have family support. Take, for instance, the women who come from foreign places. They don't have family here, so to get out and be amongst other people, that alone can help your mental health" *Focus Group 2:* "I always feel like they need to be a black NCT group because I never joined it because I didn't feel like it would be suitable for me, but if there was a black version where I could meet like-minded mums that have some shared experiences so like…it’s very different to having a white son, and having a black son cause we have different things that we are worrying about." *Interview 2:* "the lack of community and the lack of support is actually causing a lot more issues where women are feeling more and more isolated, feeling more and more alone and despondent." *Interview 3:* "we find that parents go and meet each other - it’s a big thing ... it's sharing your journeys together and realising that somebody is going through the same thing as you and you might find that you do something outside of that as well. That’s so important to be involved in." |
|  | **Engaging in varied and creative activities** | *Focus Group 1: "*when we listen to songs and the words that are in the songs, there’s a healing behind it, there’s emotion behind it. And it kind of allows you…it forces you to recognise that. If you need not cry, you cry." *Focus Group 2:* "I found music did help me a lot. You know, I'd put on my music. I'd be dancing and, you know, holding the baby and, swinging the baby around" *Interview 2:* "When people are going through an immense level of stress, singing definitely helps to calm the soul, calm the body down and put the body in a state of rhythm which is so nurturing and it's undeniable." *Focus Group 3:* "I didn't want to talk to people or be around people and so I think not having the pressures of interaction with others, that's like organised fun, will mean that you can have some type of outlet in your own setting. Filtered for yourself or specific for yourself." *Focus Group 4:* "I loved listening to music. Music was always my kind of outreach for just relaxing or just sitting in the park and grounding my feet on the grass when the weather was good." |
| **Theme 2: The overwhelming pressures and expectations of motherhood** |  |  |
|  | **Feeling consumed by the transition to motherhood** | *Interview 2:* "it's very easy to just become delusional when you're going in and out and your routine is completely changed ... it can be an adjustment to the crying and the nappy changing and the feeding and staying in the same place because you're breastfeeding potentially or having stress." *Interview 2:* "I don't think we're good at being OK with not knowing. I think the fear of that is really crippling a lot of people sadly in this and it's causing a lot of anxiety with wanting to know how am I going to do this? What's this birth going to be?" *Focus Group 3:* "Even though some people have wonderful experiences, it's all you know, pink and rosy and textbook. The reality is it’s not, and that's OK. I know for myself, when I didn't do certain things I was like, oh, and then you end up creating more harm than good, because you see what is expected. Therefore you try to attain it. You don't meet it. And then you're in a spiral. And so I think just being realistic really helps." *Interview 3:* "We're not looking after ourselves no more, so we're on our tip toes. We might be in the bath and we hear the baby cry and we're having to run out to breastfeed and we don't have to, but because we're trying our best to be the best parent. It's very, very overwhelming." *Interview 4:* "it's fine to grieve your old life, because it's a massive change to have nobody dependent on you and just being able to get up and go about your daily life to suddenly having someone solely dependent on you." |
|  | **Mentality of perseverance and resilience** | *Focus Group 1:* "A lot of people don’t want to feel judged … that’s why they don’t share, and why they always think they need to put on a facade, because they feel like it’s easier doing that, and they feel like they can cope." *Focus Group 1:* "I guess for me as well, culturally, Black women don’t go to these kinds of groups essentially. You know, you kind of just stay at home, get on with it, look after the other kids, and if people come round and help you that’s a bonus." *Interview 1:* "Especially culturally, a lot of women of colour or ethnic minorities are expected to just get on with it and it's a blessing you had a child, you shouldn't be miserable - “the baby blues” as some people call it. In some cultures, they don't even call it that, they just tell you to get on with it." *Focus Group 2:* "We are told: “We are the strong black women. We must hold everything in. We must get along with it because we're strong” No! We should not be strong. We should be able to talk about what we need to talk, same as a white person should be able to talk without judgement, you know, without any preconception, misguidance, whatever word you want to use. But as you know, we're living in a white society there. There is this judgement for black women, then."  *Focus Group 2:* "What can help I think is about knowing the strategies beforehand, so techniques and things that you can do day to day. So different people do different things: some people put on one of those apps, like CBT training apps, where you can try and change your thoughts and how you're feeling." *Focus Group 4:* "Sometimes I just had to grin and bear it and just get on with it. It's like what doesn't kill you makes you stronger, right?" |
|  | **Impact of perinatal life and health stressors** | *Focus Group 1:* "I also had something called hyperemesis, which is like debilitating sickness throughout my whole pregnancy. So I spent a huge majority of my time just in a dark room in bed, vomiting, curtains closed and not being able to eat or function. And that had a huge effect on my mental health because I was depressed." *Focus Group 2:* "I think where I probably experienced stress was after giving birth to my daughter…she technically died at childbirth and had to be resuscitated ... I had a traumatic birth, passed out, ended up in A&E and then I think it was more the stress and the trauma of having to wake up after giving birth and make my way to hospital everyday for a few weeks to look after a child and leave your child in the care of someone else." *Interview 2:* "I think one of the most, especially particularly now, is financial stresses and this I think can happen for women who are in the working class, because now with inflation going up, the cost of everything going up, it's added that pressure on. Wages haven't gone up. Maternity pay only slightly" *Focus Group 3:* "you might have a low mood because of the actual hormonal impacts, but then you're also thinking about your environmental impacts of maybe not being able to go out as much or not having that freedom of movement. Then you're also thinking about actually the financial implications of having a child. I think there’s such a relationship there that is often not linked, but needs to be." *Focus Group 3:* "I think [the severity of mental illness] depends on circumstance, so the type of pregnancy that's being carried. If it's a strenuous one or a risk-based one, I think that everything is heightened with that and so the mental strain deepens." |
| **Theme 3: Systemic barriers to accessing perinatal mental health care** |  |  |
|  | **Lack of recognition and normalisation of perinatal mental health problems** | *Focus Group 1:* "I didn't even realise it was postnatal depression. Again, despite my profession [as a midwife], I didn't recognise it" *Interview 1:* "Just knowing that it's not a phase or a facade, so be more advertised widely, or talked about more widely by the practitioners, the GPs, the midwives, the consultants as a normal thing, rather than it be a cliche thing, or you see the mother is struggling and then you mention it… because just to be open and honest" *Focus Group 3:* "In terms of the community of mothers, your mother didn't talk about it ... you then don't talk about it, because everyone that has had kids that you know hasn't mentioned these things and therefore maybe they wouldn't get it." *Focus Group 3:* "You can see those who are now having kids and be like, oh, that's what that was and they can articulate that, so I think there's more awareness then there was, nine years ago, in as much that there's a conversation that is in the air, so we can start having those conversations." *Interview 3:* "feeling that no one wants to help or support you, or you find that people are like, no, it’s not postnatal depression, depending on your background and your culture" *Focus Group 4:* "They're not interested in what you want or how to help you attain the best outcome that you need. They are more concerned with the health aspects of it, not so much the mental health aspect of it." |
|  | **Negative and impersonal healthcare experiences** | *Interview 2:* "we need every woman who has had a baby or who is pregnant needs to have recurrent touch base appointments with a mental health person or a counsellor at least once every couple of weeks. It’s so important, because even after you've had a baby, your first appointment with the GP is 6 weeks later, right? And even then, they're asking you a whole series of questions, because it's very clear they want to get you out of the door as soon as possible." *Interview 4:* "I want that woman to know that I care enough to do something about it with her rather than just leave her to get on with it, because it's her care. It's not a tick box exercise to me. Her care and feeling well is in my best interest to know that I've done a good job and that she feels safe and empowered enough to continue with her day-to-day life in existence." *Focus Group 4:* "I had a C-section, so you're not really that mobile, so to be told like, “Come on, you need to get out. Come on. We've got to go”, every morning. It was just awful, and then you see the way that they're treating other people. I saw this other lady, I think her scar must have opened, her wound opened up and she was bleeding and she couldn't move and they were like “Look around you, it's a state, why are you not getting up? Why are you not tidying up yourself?” rather than providing the support that she needs" *Focus Group 4:* "Teach people the right way, and sometimes people only learn by doing. I'm not a theoretical person, I'm a practical person, so you know everyone has different learning styles too, so it's to put all of those practicalities into place and work on the best plan for that specific person, not treat everyone the same." *Focus Group 4:* "when I first turned up at the ward that night and I gave them my birthing plan and the midwife or the consultant that was on that night. She was like, “oh, no, I don't need to read that. That's something for you to do to keep you occupied.” Yeah, they didn't read it, but everything I did not want they did." *Focus Group 4:* "They were trying to put me in for a C-section because I already had a C-section. But I said “No, I can have a natural birth", because it wasn't like an emergency situation. It was a planned C-section I had before, so I knew my rights. ... And they were quite angry with me, like the nurses were like, “I've never heard of anything like this before” they were nasty, they were just so awful to me." |
|  | **Distrust of healthcare systems and staff** | *Focus Group 2:* "I found speaking to other parents that some of them did have suicidal thoughts, and you can't speak to nobody about your suicidal thoughts, because the first thing a health visitor is going to do is call the social services to come and take away your baby. So they had to suppress all these feelings" *Focus Group 2:* "We should be able to talk about what we need to talk, same as a white person should be able to talk without judgement, you know, without any preconception, misguidance, whatever word you want to use. But as you know, we're living in a white society there. There is this judgement for black women, then."  *Focus Group 2:* "I would say probably for a lot of women, there is that reluctance to go to a professional such as a GP or health visitor just because of that fear of being judged and when you do intersect it with being a black woman and having to feel like you have to be the strong black woman, I think often we do suppress and just get on, then probably by the time we have gone to someone, even if it's a partner or a family member, we're probably feeling really bad." *Interview 2:* "But I for one can say, even as a healthcare professional after having a baby, I didn't feel like I could go to my own GP and talk about some of the issues that I had." *Focus Group 3:* "there's no way I'm gonna go and talk to my GP or health adviser and say I'm feeling depressed. They're gonna take my baby away. They're gonna think I'm not capable, so I think seeking professional help is not something people out rightly will lean towards." *Focus Group 4:* "I find it easier to speak to somebody who's not family than to speak to family. Family will always say, “oh, don't be silly. It's not that, it's blah, blah, blah”. And not actually take your concerns into consideration. Whereas somebody who's trained within that field, I would hope, would be able to talk to me about what my options are." |
| **Theme 4: Suggestions for future perinatal mental health music-based support groups** |  |  |
|  | **Varied priorities and preferences for activities** | *Focus Group 2: "*So I think for me as a non-singer, I wouldn't want to go because now I'm giving myself an extra thing to be anxious and worried about, so if it was like a group setting or even in front of someone I don't know, I would just cringe ... So I think for me it wouldn't - as much as I love music and I know lots of songs - it would be a turn off for me." *Focus Group 3:* "I mean, every time I've done karaoke with people that I don't know, I've had a great time. I don't know them, but we've picked songs and they like their song and you big them up. The energy's right. So I think that kind of singing is great" *Focus Group 3:* "just like a singing space, whatever genre of music, where you can just proclaim. Just be free." *Interview 3:* "I would also say writing would be a powerful source, so it could be even through poetry ... if we put what's here in our minds, what we're going through on a piece of paper, it might help us understand what we're going through more deeply within us, because then we could be like, oh, this is actually something I can work on. It's expression, it's self-expression, so you might not want to sing, because people are like “I can't sing, I don't want to sing. No, this is embarrassing.” So it would be good to do it as a group and everyone just says yes - this is how I feel. Like those that are hosting it, be the first and even if they try and do it in a way that it doesn't sound so professional so that it makes them feel like OK, I can do this" *Focus Group 4:* "We used to go to the library on a weekly basis and do Bounce and Rhyme and all the rest of it. So if it's a different version of it, maybe where we can sing culturally appropriate songs, or, you know, in different languages or something that's appropriate on that sense then yeah, something that's a bit different from Wheels on the bloody Bus." *Focus Group 4:* "I was kind of thinking probably selfishly, but I've always thought it was more me-time, this is a time for me to go out and do something for me, like on my own." |
|  | **Strategies to facilitate inclusivity and accessibility** | *Focus Group 1:* "One thing I’ve found with running groups for mums is that timing is so tricky. There’s never a good time in a way, like do you do it on a weekday morning? Do you do it on a weekend, but then everyone’s doing the shopping or the kids have activities if you’ve got older kids? If you do it on a weekday, people might be back at work if they’re on a short maternity. So like if you do it in the evenings then you have all the bedtimes and maybe you don’t have childcare." *Interview 1:* "I like the idea of online. So you've not got the stress and the worry of organising your child and getting them ready. But then also, I do like in person as well. So a choice of both? A choice of both so it's not too restrictive." *Interview 2:* "I find that there's a way in which some of these groups are cultivated, not to say that they're excluding, but let's say there was a musical group that's going on. Are we cognisant of other cultures? Are we having African music, for example, and Afro Caribbean music and other cultural music? Or is it just going to be Mozart? Or a certain type of music, because even in those little nuances, we can unpick that and say, OK, this might not be for me. So it could be things like that actually cause a little bit of a division sometimes I find. Even though it might not have been intended, there's a lot to be said for when you don't say something sometimes." *Focus Group 3:* "So if I ever do an activity, it'll be between 10:00 and 2:00. I won't do anything before or after. Also thinking about drop-in. I think there's so much pressure when you're a parent, because it starts at let's say 11, and if I've missed it by 10 minutes, I'm now not going at all." *Interview 3:* "Making it local in different places so that it's accessible as well, so even if they're running a bit late, they're not very late. So just spreading it out across, because I find some boroughs get more funding" *Interview 4:* "The intimacy of the group: is it a group that has 12 people and for some people meeting a huge group is quite intimidating? Or is it a group of four to five mums and their babies and actually, that's a bit safer, feels more intimate?" |
