# Supplementary material for: Exploring the experiences of women of African, Caribbean and Mixed heritages to inform a music-based intervention for perinatal mental health in South East London: A qualitative study
Source: PLOS Ment Health. 2026 Mar 23;3(3):e0000477. doi: 10.1371/journal.pmen.0000477 (PMC13008082; doi:10.1371/journal.pmen.0000477)
Supplement: Attachment — Submitted filename: Response to Reviewers.docx [file pmen.0000477.s003.docx]

Response to reviewer comments

Dear Reviewers,

We appreciate the time you have taken to provide comments on our paper, which have enabled us to make significant improvements. We have carefully considered and addressed each comment. Below we provide a point-by-point response. Our responses to each individual comment are in blue text and we then quote the relevant changes from the manuscript where appropriate. All modifications in the manuscript have been added using track changes and are visible underlined and in colour. We have additionally uploaded a version of the manuscript with all these changes accepted.

**Journal Requirements**

1. In the online submission form, you indicated that “The qualitative data that support the findings of this study are available on reasonable request from the corresponding author. The transcripts are not publicly available to protect the privacy and confidentiality of participants, as they contain potentially identifiable and sensitive information.”

All PLOS journals now require all data underlying the findings described in their manuscript to be freely available to other researchers, either 
1. In a public repository, 
2. Within the manuscript itself, or 
3. Uploaded as supplementary information.

This policy applies to all data except where public deposition would breach compliance with the protocol approved by your research ethics board. If your data cannot be made publicly available for ethical or legal reasons (e.g., public availability would compromise patient privacy), please explain your reasons by return email and your exemption request will be escalated to the editor for approval. Your exemption request will be handled independently and will not hold up the peer review process, but will need to be resolved should your manuscript be accepted for publication. One of the Editorial team will then be in touch if there are any issues.

We have changed our data availability statement to the following and emailed PLOS to confirmed this (currently waiting on a response):

“All relevant data from the focus group and interview transcripts are available either in the manuscript or in S1 Table. In compliance with the ethical approval received from the Ethics Committee of the Psychology Department at Goldsmiths, University of London, full focus group and interview transcripts cannot be publicly shared, as participants did not consent to this and it would compromise participant confidentiality.”

2.  Please insert an Ethics Statement at the beginning of your Methods section, under a subheading 'Ethics Statement'. It must include:

1) The name(s) of the Institutional Review Board(s) or Ethics Committee(s)

2) The approval number(s), or a statement that approval was granted by the named board(s)

3) (for human participants/donors) - A statement that formal consent was obtained (must state whether verbal/written) OR the reason consent was not obtained (e.g. anonymity). NOTE: If child participants, the statement must declare that formal consent was obtained from the parent/guardian.

We have now included the ‘Ethics Statement’ under this subheading.

3. If the reviewer comments include a recommendation to cite specific previously published works, please review and evaluate these publications to determine whether they are relevant and should be cited. There is no requirement to cite these works unless the editor has indicated otherwise.

Acknowledged.

**Reviewer 1 comments**

1.1 Overview and general recommendation

*The following paper describes a qualitative study focusing on focus groups and interviews with 14 women of African, Caribbean and Mixed heritage to explore their perinatal experiences and preferences regarding music-based support to inform a culturally inclusive perinatal participatory music intervention. It describes four overarching themes and provides some future recommendations and directions for research.

Thank you so much for sharing your work and your manuscript. It has been a pleasure to read and review this paper. This paper meets the scope and partly meets the criteria for publication as it stands. There is a strong argument made throughout, and this is work that will have value for future culturally inclusive music interventions.

Thank you very much.

However, there are a couple of areas where I encourage you to further develop your ideas to take this work to the next level. Particularly, I would encourage you to look back at your methodology and results when it comes to your position statement of taking a critical realist stance. At the moment, it is unclear how the critical realist philosophy has been woven into the work and themes, which creates a misalignment. You will see many of my comments are about what you say you have done vs the critical realist stance you say you have taken. You could do constructivist or critical realist for this work, both appear appropriate. But whatever you choose, it must be consistent throughout.

Thank you for your very useful comments on the critical realist stance. Upon reflection, we are in agreement that the constructivist approach is more appropriate for the aims of our study. Nonetheless, we were very interested in your thoughts around the critical realist approach and are grateful to you for engaging with this aspect of our work in such detail. The points raised were very useful for deepening our understanding of both approaches and refining our methodological position.

There is also a possibility to move beyond description in your results and engage a little further with the data. Please see specific comments below that hopefully should be able to help and guide the revision, all in the good spirits to advance this already great piece of work.

We have reviewed the results section and added in more interpretation within each theme to engage further with the data (see section 2.4 below and the manuscript for more detail on the changes made).

2 Major comments

2.1 Title and abstract

*In the title, you rightly mention that it is a qualitative study. Is there a reason why you don’t mention that this is a critical realist qualitative study? I am just a little confused as to the stated philosophical position and the lack of engagement with that philosophical position throughout. If the intention is to go through a constructivist lens (which currently reads more as such), then that is absolutely fine, but I wonder how much critical realist you are adding or not adding.

As per our decision to go down the constructivist route, we have not made any changes to the title.

* You mention in the abstract (line 24) that inequalities occur at different levels of the system, which necessitate a greater reliance on self-coping strategies. It might be helpful to clarify in the introduction how self-coping strategies operate differently across these levels, or why they may be more effective at some levels than others, to guide the reader and position the music intervention within one/various layers of the system you mention.

We decided that mentioning self-coping strategies in the abstract diluted and confused our focus on participatory music-based interventions, which are not necessarily self-coping strategies in themselves. Therefore, we have removed the clause referring to self-coping strategies to ensure a streamlined focus on the role of community music-based interventions.

*In line 35, you highlight the individual experiences of the perinatal period, which is absolutely fine, but maybe through a critical realist stance, you may want to share the dynamics between structure and agency and some of the repercussions that have on creating culturally inclusive interventions.

As per our decision to go down the constructivist route, we have not made any changes to this part of the abstract.

2.2 Introduction

*From lines 59 to 64, you mention some great information that from a critical realist stance can be very useful (i.e. systemic issues, looking at the problem from a more emancipatory lens, critical analysis of power relationships and value-laden aspects of science…). However, at the moment, it is placed as background information. If you are going to work on the critical realist stance, make sure that you align your wording and examples to reach philosophical coherence.

As per our decision to go down the constructivist route, we have not made significant changes to this section as we believe it already aligned with our philosophical position. However, we have slightly changed the wording:

“Evidence emphasises the systemic nature of ethnicity-related inequalities in statutory mental health services due to “monocultural and reductionist frameworks of assessment and treatment” and “direct experience of racist practice” [17, p.1]. These wider social and cultural contexts are reflected in and interact with the individual experiences of Global Majority women, where prevalent barriers include language and communication challenges, stigma, mistrust of healthcare services, cultural expectations and family influences [18-21].“

*There is a good discussion from lines 79-91 around community-based services, which is well referenced and justified. However, it then moves to interventions, which are slightly different to services. I.e. building culturally inclusive services and interventions. I think the confusion lies here that you are interchangeably using in these lines the words services, interventions and programmes. It would be good to keep consistent in language to not confuse the reader as to whether you are talking about services or interventions, and make sure to build the gap as to the need to work on both.

We have now ensured consistency with the terms services, interventions, and programmes throughout. The third paragraph (lines 65-80) focuses on healthcare and community *services*, building the gap for the need to work in community settings, and the fourth paragraph (lines 81-93) explains the importance and benefits of community-based *interventions*.

*For your research questions and text beforehand, it reads very much from a constructivist lens. Again, no problem if that is your intention, but there is a philosophical misalignment here between the needs of a critical realist approach, which is beyond experiences to the powers, underlying mechanisms, structure vs agency and other concepts in comparison to more constructivist experiences.

As per our decision to go down the constructivist route, we have not made any changes to our research questions.

2.3 – Methods

*Is there a rationale as to why some participants did focus groups and some others did interviews? What are the potential implications of this decision? As well as the numbers of each, having four focus groups of smaller numbers rather than two or one of larger numbers?

The small size of the focus groups and combination of focus groups and interviews was a result of logistical difficulties, which led to the focus groups having smaller numbers of participants than intended and sometimes becoming interviews.

*You may want to add a reference or justification as to why your sampling techniques were the most appropriate to meet your study aims.

We have now included the following in the main text:

“This sampling method ensured efficient recruitment of participants who matched the specific demographic required for our study. In addition, their connection to TA meant such participants might feel more comfortable talking about their experiences of the perinatal period.”

*Could you elaborate on the rationale for using the same questions in both the focus groups and the interviews? Given the distinct purposes and interactional dynamics of these methods, it would be helpful to understand how this decision supported your study design.

Our questions for the interviews and focus groups were similar to enable us to focus on eliciting experiences related to the research questions in both settings, while flexibility in how they were administered ensured that each interview or focus group was reflective of participants’ lived experiences. We have addressed this in the text with the following:

“The semi-structured nature of the focus groups and interviews allowed similar questions to be asked across the participant group to align with the research questions and aims of the study. Nevertheless, the question guide was used flexibly alongside follow-up questions to delve into participants’ responses and enable women to share the meaningful aspects of their lived experiences. TA also tailored the questions to the different dynamics of the interview and focus group settings, including more guided reflection on the commonalities across participants’ experiences in the focus groups.”

*Is there a reason why the questions used did not directly address the cultural relevance of the interventions? As some of the questions are broader for “women” (such as question 6), was there clarification that this was to build culturally relevant interventions as well?

We decided to not ask explicit questions around cultural relevance as participants were already made aware that the study was aiming to explore their specific experiences as women from African, Caribbean and Mixed heritage backgrounds and to devise ideas around culturally relevant interventions. This has been clarified in the main text:

“Participants were made aware of the study’s intention to explore ideas around culturally relevant interventions on the information sheet and at the beginning of the discussion.”

*The biggest concern here is lines 197-201 and the description of critical realism as the philosophical stance. I would encourage you to use some references here, explain to the reader what critical realism means and what the implications are of adopting such a lens for your study beyond the description you currently provide. For instance, there is no mention of depth ontology, ontological realism, power dynamics, etc. There is a slight comment on agency vs structure, but it is not substantiated enough, i.e. could add Archer's morphogenesis, for example, here. It is a philosophical basis rather than a theoretical one. This is not a methodological paper, so I wouldn’t expect to see a full description, but it is an important claim made that then impacts the analysis, and, as mentioned previously, I don’t see how the critical realist stance informed your results for now, as they read more constructivist. For instance, there is no mention of mechanisms, retroduction or other critical realist language I would expect to see.

As per our decision to align our paper with the constructivist approach, we have adapted this section to include the following:

“The analysis was conducted using the philosophical underpinning of constructivism, which acknowledges that each individual’s reality is shaped by both subjective perspective and past lived experiences in interplay with social interactions [45,46]. This theoretical basis enabled the analysts to consider individual meanings of each participant’s experiences as a reflection of the wider social and cultural contexts within which their realities were formed, and used this to explore inequalities at play on a personal, social, and systemic level for each participant [47]. The design choice to have a local community leader with shared Global Majority heritage to lead the focus groups and interviews enabled participants to discuss past experiences of the perinatal period while sharing ideas related to broader systemic inequalities; through a constructivist lens, analysis considered the way that knowledge was thus co-created within the focus group and interview spaces [48].”

2.4 – Results

*My main observation in the result section is twofold, and this is relevant for all sections and themes, hence it all comes in one paragraph. Firstly, there is a lot of great descriptive content that shares ideas around the themes, but it remains superficial at times and does not engage deeply with the depth ontology or mechanisms, which, if using a critical realist stance, I would expect to see. For example, in 1.1, there is a description of participants' value on family and friends, others expressed challenges, and others felt isolated. The content you could consider here (and throughout the other sections) is – so what? What is it about having critical relationships or being domineering that helps us understand the needs of the culturally inclusive music interventions better? In the discussion, you go into more detail, but it would be good to expand the results with more interpretation and analysis, rather than description.

We have reviewed the results section and added in more interpretation to engage further with the data. We have analysed the quotations and other data from the transcripts in more depth to provide additional interpretive insights about participants’ experiences. For each theme, we also considered ‘so what’ to further explore their meaning in relation to a future culturally inclusive music-based intervention for perinatal mental health. Please see the manuscript for the specific changes made, as there are too many to document here.

The second element in the results is (sorry for sounding repetitive) the critical realist stance. I don’t see the philosophical alignment. You mention relationships between individuals and contexts more widely, but there is a lack of a narrative that aligns with the aims of critical realism. I encourage you to look at this section and 1. Increase the analytical depth and 2. Align more with the critical realist stance you took to showcase how this philosophy of science impacted your analysis and results. There is good work by Wiltshire, Ronkainen or Fryer on how to do Realist Thematic Analysis. I see this is not what you did here as described by these authors, so there is no expectation to do the same, but it may give you some ideas on some of the wording or things to include or indeed consider the philosophical stance.

Having decided that our philosophical position aligns more with constructivism in this paper, we did not make any changes to the narrative specifically to align with critical realism. However, we did review the paper by Wiltshire and Ronkainen, which helped us to consider what to include to increase the depth of analysis and interpretation.

2.5 - Discussion & Conclusion

*This section may change slightly based on the thoughts above on how you decide to weave (or not, if going for an interpretivist lens) the critical realist lens. For example, in the first paragraph, do you have any thoughts on structure vs agency on that particular point of systemic ethnicity-related inequalities in mental health care? There is a great book coming soon on this (Decolonizing Global Health: A Critical Realist Perspective).

As we are following a constructivist lens in this paper, we did not make any changes to weave in a critical realist perspective. However, we did expand a few elements of the discussion (see section 3.5 and the manuscript for details).

*In your limitations section – look at the comment below in minor comments around language. I would also encourage you to move away from saying that your study is non-generalisable. That is not a limitation of a qualitative study, after all, it has different rules and assumptions about generalisability than quantitative work, and the aim is not to generalise. But you may want to talk about transferability (or theoretical generalisability instead of statistical generalisability) instead, as a concept, which appears more appropriate for this research.

We have removed the mention of generalisability in the limitations section and focused on highlighting the need for other context-specific research in future.

3. Minor comments

3.1 Title and abstract

*In line 30, you can be more specific as a perinatal mental health participatory music intervention, as then you differentiate between perinatal care more widely and perinatal mental health. You can do the same in line 34, suggestions for future perinatal mental health creative support groups. Have a look throughout the manuscript to see where you could be specific about perinatal vs perinatal mental health care. I have seen a few examples throughout.

We have addressed this in the main text to ensure that ‘perinatal mental health’ is specified where necessary to describe the intervention.

*There is again no mention of the critical realist position taken in line 31, described as an inductive reflexive thematic analysis. You may want to add informed by a critical realist stance or philosophy, and then explain more later on how that has impacted your interpretation of results.

As per our decision to go down the constructivist route, we have not made any changes here.

3.2 Introduction

*Line 51, you may want to spell out a couple of what those healthcare barriers may be.

We addressed this by including the following: “such as healthcare professionals dismissing concerns or normalising symptoms”.

*Lines 75-78, a very good example of the WHO's need to move towards community-based. Any UK-based examples you want to share, i.e. NHS 10-year plan or similar, to position the need from a UK perspective too?

We have addressed this comment with the addition of:

“In the UK, the 10 Year Health Plan for England [26] also explores the need to improve the accessibility of healthcare by engaging with communities, including through integrated neighbourhood teams, which aim to connect residents with various healthcare, social care and voluntary organisations tailored to their health needs.”

*You use the word mechanism in line 93. If you are using a critical realist stance, be careful with the wording of the mechanism and how it will be defined, as in critical realism, these have a very specific meaning.

As we are not using a critical realist stance, we have not made any changes to our definition of mechanism.

*In lines 107-108, you highlight “little consideration about how musical activities can be integrated into inclusive community groups”. This is an implementation question, not an intervention question. Your aim in the abstract is “inform a future culturally inclusive perinatal participatory music intervention”, not necessarily about how to integrate them. We need to know what they are first, their components and their effectiveness before we can fully integrate them. Therefore, you may want to consider rewording this sentence to focus on the intervention, rather than the implementation, to meet the needs of the study.

We have addressed this comment by using the following wording:

“there has been little consideration of how inclusive perinatal mental health music-based interventions can be developed with and for diverse women in the UK”

3.3 – Methods

*You use fourteen in line 145 but 14 in the abstract. Remain consistent as to which one you are using.

We have now ensured consistency throughout.

*You mention in the inclusion criteria in line 146, “women who have given birth to at least one infant”. Does this mean that the women interviewed or included could have had an older child and have already gone through the perinatal period? Just to clarify for readers to understand if women could have been experiencing the perinatal period at the time of the interview, or retrospectively share their experiences.

We have addressed this by including the following (lines 174-175):

“We invited women to retrospectively reflect on their experiences of the perinatal period, and none of the women were in the perinatal period at the time of study.”

*In Table 1, it could be useful to highlight or share how each question meets the five topics shared in the preceding paragraph, maybe at the end of each sentence with a (1) or similar, to showcase how each question aligns with the topics.

We have included numbering to relate the guide to the topics as suggested.

3.4 – Results

*Figure 1 – You may want to make the distinction between the different themes and subthemes clearer, maybe through colour coding each theme and subtheme or through labels?

We have put the themes and subthemes in different colours and shades to make their distinction clearer.

*Is there a reason you mention creative support groups rather than music-specific ones? That appears as more general rather than specific to the music needs of the intervention you are trying to co-develop. Of course, music is creative, but is it under that umbrella, and it shows that you want to tackle the music specifically.

We have changed ‘creative’ in Theme 4 to ‘music-based’. However, we have kept the term ‘creative’ in Subtheme 1.3, as this subtheme is referring more broadly to different activities, such as music, dance and journalling.

*There is a lot of information on barriers and service use (or lack thereof) for the participants, which is important contextual information. However, I see that the cultural elements are less emphasised throughout, except in a couple of elements. As this is a specific study looking at culturally inclusive music-based interventions, I wonder which of the results you suggest are either specific to cultural inclusivity or wider problems with healthcare services and dynamics. It may be worth considering this to really engage with the question.

We have engaged further with the cultural elements of participants’ narratives at several points in the results section (see manuscript for additions). To address the question of which themes are specific to cultural inclusivity or wider problems, we have also added several sentences at the end of the first paragraph of the Discussion section.

“Many of the systemic issues with perinatal mental healthcare evident across participants’ experiences in this study appear common to women from other backgrounds and contexts. Similarly to our findings, other research has suggested that barriers to perinatal mental healthcare across the UK include being scared of social services, fear of judgement, lack of social support, limited awareness and previous negative experiences of healthcare [10]. However, culturally-specific expectations, experiences of stigma and mistrust played a significant role in heightening the negative impacts of various barriers to perinatal care. This could explain the prevailing perinatal health inequalities associated with Global Majority ethnicities [12,13]. These experiences are further explored below, with an emphasis on how they can offer specific suggestions for future culturally inclusive music-based interventions.”

3.5 - Discussion & Conclusion

*Line 447 – if using word mechanisms and a critical realist stance, be careful in how mechanisms are worded and conceptualised throughout. Mechanisms, as per Mingers and Standing (2017) through a critical realist lens, are “a structure of inter-related parts together with the powers or tendencies that the structure possesses”. Or can see Bhaskar's work for his conceptualisation. The point is to be clear about what definition you are using, so that there is alignment in what you mean.

As we are not using a critical realist stance, we have stuck with the general meaning of the term ‘mechanism’ as a process. We hope this is sufficiently clear for readers as a general term, so we have decided not to add a definition.

*The strong social connections paragraph is well developed, but is there value in positioning this within the music intervention per se too?

We have added the following sentence to this paragraph to position the need for strong social connections within the music intervention:

“Indeed, music-based interventions could align well with this need, due to their ability to foster a sense of belonging, group connection and social support [30].”

*The conversation on language and stigma is really interesting again from a critical realist stance in lines 480-489. I am not sure if you have already, but Braun and Clark, in their recent book, have a section on this, citing the work of Maxwell. You may find it interesting to develop these ideas further.

We explored the section in the Braun and Clark book and their citations of Maxwell, but decided not to include these as they focus on the critical realist stance. However, we did explore some other literature around stigma and language, which pointed us to some further suggestions around building partnerships between healthcare services and diverse communities – we included these ideas at the end of this paragraph.

*Fig 2. May want to make the title specific to music interventions rather than community perinatal mental health support interventions, as there could be many! You may want to comment in the recommendations for future music-based interventions about implementation science and how this research could also be used to help move us from not creating an intervention only, but also to achieve health equity through implementation science approaches.

We have made the title of Figure 2 specific to music-based interventions. We have also added the following sentence at the end of the recommendations paragraph to comment on potential implementation science approaches:

“In addition to intervention development, this research could also guide inclusive approaches to the implementation of future participatory music-based interventions.”

I hope this review is helpful and clear, all in the spirit of advancing scientific knowledge and supporting you in taking this important work to the next stage. Thank you for your efforts!

Thank you very much for taking the time to review our manuscript!

**Reviewer 2 comments**

The Introduction should conclude with an explicit problem statement, as the current formulation remains implicit and may leave readers unclear about the study’s precise focus.

Thank you for your suggested revisions. We have included the following sentence to ensure clarity for the end of the introduction:

“In doing so, our study addresses a significant gap within current psychological research around the needs of Global Majority women during the perinatal period and possible supportive mechanisms, including music, which is pertinent due to the increased maternal death and morbidity rates for this population.”

Additionally, several parts of the Introduction reiterate similar ideas about healthcare inequities and mistrust; consolidating these points into a more streamlined paragraph would improve readability and coherence.

We have kept the same structure in the introduction, but have tried to remove or combine sentences where they reiterate similar ideas about healthcare inequities. In particular, in the third paragraph, we removed the first sentence, which was reiterating earlier points, but kept the sentence about systemic barriers and mistrust in lines 69-70 as we felt this was needed to justify our focus on community-based interventions.

In the Results section, some subthemes rely heavily on lengthy participant quotations. Shortening these excerpts and expanding the accompanying analytical narrative would help achieve a stronger balance between interpretation and evidence.

We have reduced the length of several quotations and expanded the depth of the accompanying analytical narrative of each subtheme. By including additional analysis of the data and interpretation of participants’ experiences in relation to future interventions, we hope we have now achieved a stronger balance across the results section. Please see the manuscript for details of the various additions made.

The Discussion would also be strengthened by more consistently linking each theme to existing research, ensuring that the findings are situated within broader scholarly conversations.

We have added several references to the discussion section to ensure each theme is more consistently linked to broader scholarly research – please see the manuscript for the specific additions.

Finally, the coding process needs more detailed description. Specifying the steps taken (such as line-by-line coding, independent coding by multiple analysts, and iterative refinement of codes and themes) will allow readers to fully understand and assess the robustness of the thematic analysis.

We have included an initial sentence to summarise the coding process before going into detail:

“Line-by-line coding was initially carried out by each analyst independently, before collaborating to iteratively refine the codes and themes.”

Thank you very much for taking the time to review our manuscript.
